# Helical opto-thermoviscous flows drive out-of-plane rotation and particle spinning in a highly viscous micro-environment

**DOI:** 10.1038/s41377-026-02303-8

**Published:** 2026-05-11

**Authors:** Fan Nan, Weida Liao, Adrián Puerta, Josephine Spiegelberg, Elena Erben, Ralf Mikut, Stephan Allgeier, Martin Wegener, Eric Lauga, Moritz Kreysing

**Affiliations:** 1 Institute of Biological and Chemical Systems-FMS, Karlsruhe Institute of Technology, Eggenstein-Leopoldshafen, 76344 Germany; 2 Department of Applied Mathematics and Theoretical Physics, University of Cambridge, Cambridge, CB3 0WA UK; 3 Institute of Nanotechnology, Karlsruhe Institute of Technology, Eggenstein-Leopoldshafen, 76344 Germany; 4 Institute for Automation and Applied Informatics, Karlsruhe Institute of Technology, Eggenstein-Leopoldshafen, 76344 Germany; 5 Institute of Applied Physics, Karlsruhe Institute of Technology, Karlsruhe, 76131 Germany; 6 Present Address: Department of Mathematics, Imperial College London, London, SW7 2AZ UK

**Keywords:** Optical manipulation and tweezers, Optofluidics

## Abstract

Contact-free and object-agnostic three-dimensional (3D) rotation remains a challenge at both the micro and nanoscale, with broad relevance to advanced imaging, biology, microrobotics, and materials science. Specifically, precise 3D rotation is desirable in diffusion-suppressing environments, where conventional micromanipulation methods fail. Here we introduce an opto-thermoviscous strategy that scans a focused laser spot within a two-dimensional plane to robustly generate 3D helical thermoviscous flows (TVFs) within highly viscous media. We further report on the discovery of opto-hydrodynamic focusing that converges a spiral motion to a defined particle height. By exploiting symmetry relations, we use rational design to decouple out-of-plane rotation from lateral displacements, leading to stable spinning with positional fluctuations below 200 nm, and demonstrate compatibility with a broad range of microstructures, from nano-printed tiles to stained biological cells, and even perfectly round homogenous spheres. Finally, leveraging the kinematic nature of thermoviscous manipulations, we demonstrate how stepwise rotation, alternated with 3D volumetric microscopy, can be combined with established multi-view image fusion strategies to increase resolution in biological imaging. Conceptually, this elevates TVFs from planar transport to symmetry-engineered volumetric actuation, delivering robust, material-agnostic, out-of-plane rotational control and sheathless opto-hydrodynamic focusing for all-optical micromanipulations and augmented microscopy.

## Abstract (web summary)

Focused laser scanning generates symmetry-engineered 3D thermoviscous flows for contact-free, object-agnostic out-of-plane rotation and sheathless opto-hydrodynamic focusing in highly viscous media, enabling stable micromanipulation and enhanced volumetric imaging.

## Graphical abstract

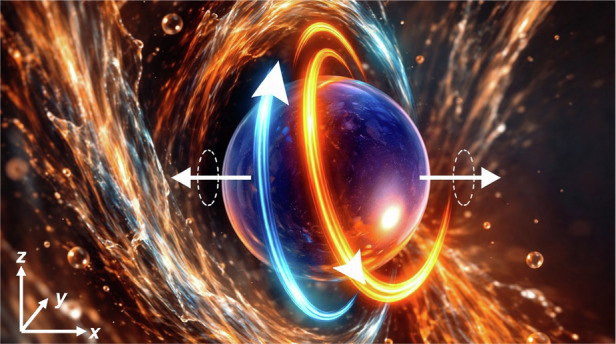

## Introduction

Precise manipulation of microscopic objects is essential across a wide range of scientific and technological domains^1–22^, including materials assembly^16,23,24^, microfluidics^2,9,15,25^, cellular biology^3,10^, and optical imaging^5,17,26,27^. In particular, the ability to impose transverse torque (thereby enabling continuous out-of-plane rotational motion) is of both fundamental interest and practical importance. It underpins applications such as multi-view microscopy, which addresses the intrinsic anisotropy of three-dimensional (3D) optical imaging, where the axial resolution is much lower than the lateral resolution^28^. While the transfer of photon angular momentum orthogonal to the beam propagation direction is theoretically feasible in some applications, achieving steady rotation remains inherently challenging. This is often limited by the requirement for samples with tailored optical properties and reduced symmetries and/or by the necessity of specially engineered structured light^29–36^. Acoustic fields offer a more accessible route to transverse torque^37–40^, but their utility is often limited by insufficient reconfigurability and reduced effectiveness at small scales. Similarly, the use of magnetic fields is constrained by challenges in scalability and spatial resolution, as well as by the intrinsic magnetic properties of the manipulated objects^10,41,42^. Very recently, multi-physical coupling strategies, such as the opto-thermoelectric rotation technique, have been developed to drive out-of-plane rotation of single micro/nanoparticles using simple, low-power optical setups^17,43^. However, these force-based methods exhibit limited effectiveness in high-viscosity environments and frequently require complex sample chambers. An alternative route lies in exploiting hydrodynamic focusing effects^44,45^, where flow fields can concentrate and direct particle motion, potentially offering a robust mechanism for stable out-of-plane rotation.

In recent years, thermoviscous flows (TVFs), generated by scanning a mildly heating laser beam through fluids, have been established as new method for precision manipulations^2,46–51^. Unlike traditional opto-hydrodynamic manipulation methods based on optical trapping, which need particles to act as handles or as actuators to generate flows^9^, TVFs can be efficiently generated in high-viscosity media without this requirement. Although previous studies have systematically explored TVFs for in-plane particle transport and even feedback-controlled force measurements^48,52^, their potential for controlling another critical degree of freedom, i.e., out-of-plane rotation, could not yet be reached. Demonstrating robust and particle-agnostic out-of-plane rotation, ideally free from Brownian fluctuations, in combination with *z*-stack imaging, is essential for achieving true multi-view information in a single-objective trapping platform, providing a powerful means of isotropic resolution enhancement without complex multi-objective designs.

Here, we demonstrate stable, tunable out-of-plane rotation and spinning of microparticles in highly viscous media using 3D helical TVFs generated by scanning a single laser spot in a 2D plane. Using line-by-line scanning, in-plane TVFs are rendered unidirectional or cancel upon scan reversal, whereas out-of-plane components accumulate, producing controlled 3D TVFs that organize into transversely propagating helices or localized vortices perpendicular to the scanning plane. Intriguingly, we observed an opto-hydrodynamic particle-focusing effect, which we apply in order to achieve simultaneous control over particle transport, out-of-plane rotation or spinning, trapping, and assembly. These 3D helical TVFs also integrate seamlessly with two-photon lithography, establishing a new paradigm for light-driven, cascaded 3D microfabrication and multiaxial rotational manipulation. Furthermore, operating in a highly viscous medium, this approach effectively suppresses Brownian fluctuations and enables 3D imaging of microstructures with improved resolution, including biological cells, as we showcase via the integration with spinning disk confocal microscopy. Proof-of-concept experiments on HCT116 cells with fluorescently stained nuclei revealed that while conventional imaging shows only one nucleus, TVF-based fusion of multi-orientation *z*-stacks instead resolves two distinct nuclei, overcoming *z*-axis resolution limits.

## Results

### 3D helical TVFs induced by 2D laser scanning and quantified by single-particle probing

**Fig. 1:**
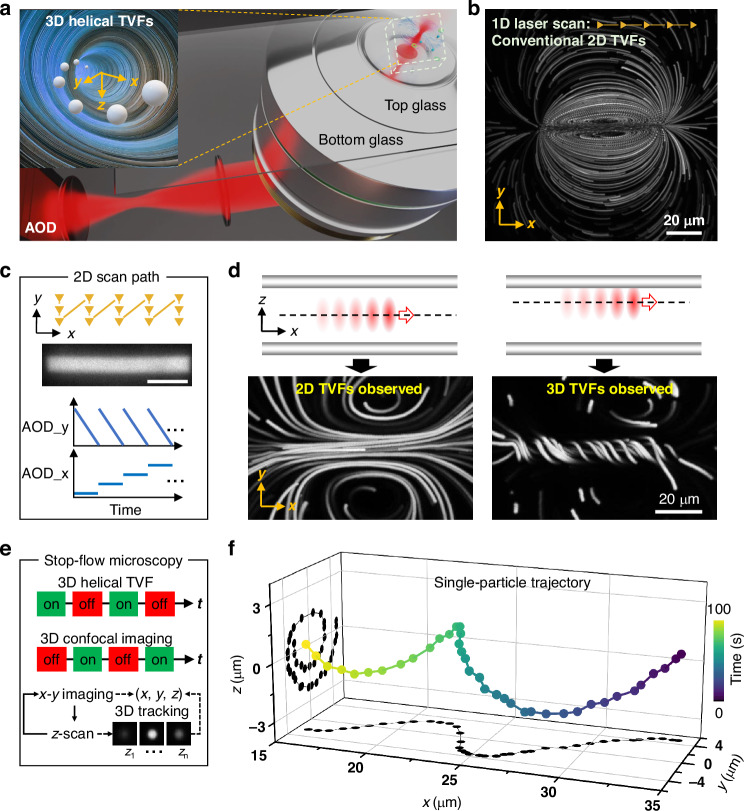
Laser-induced 3D helical TVFs generation and characterization via single-particle tracking. **a** Schematic of the experimental setup: a mildly heating infrared laser beam is rapidly scanned across a fluid sample. The inset conceptually depicts the resulting helical flow profile. **b** Maximum intensity projections of fluorescent particles over time reveals conventional 2D TVFs (in-plane) induced by repeatedly scanning the laser along a single line. **c** Schematic of the 2D line-by-line laser-scanning trajectories, which create fully 3D helical TVFs. The laser scanning is controlled by the acousto-optic deflector (AOD). The measured time-averaged intensity profile (shown in grayscale) corresponds to the rapid 2D scanning of the focused laser spot, recorded with an exposure time of 500 ms. Scale bar: 20 μm. **d** Illustration of the sample chamber cross-section, assembled from two identical glass coverslips, with the laser scanning plane positioned either at the chamber mid-height (left) or near the top glass surface (right), where the latter configuration leads to the observation of pronounced helical flows (shown in the lower panel). The chamber thickness is 43 μm. **e** Workflow of the stop-flow microscopy used to measure the 3D trajectories of probe particles. **f** A representative measured 3D single-particle trajectory reveals that the 3D TVFs contain a helical structure that simultaneously transports and rotates the particle. The scan area spans approximately 68 µm in the *x*-direction and 7.6 µm in the *y*-direction, discretized into 400 steps in *x* and 50 steps in *y*, resulting in 20,000 scan points per full scan cycle. The laser scanning frequency is fixed at 500 kHz, which corresponds to an overall scan repetition rate of 25 Hz for the complete scan pattern

Figure 1a presents a schematic of our experimental setup alongside an overview of the 3D helical TVFs, which enable simultaneous rotation and transport of particles. In this configuration, an acousto-optic deflector (AOD) rapidly scans a moderately focused and mildly heating laser spot (to provide local heating by a few Kelvin) within a 2D plane, facilitating the generation of 3D TVFs. Previous studies have primarily focused on in-plane 2D TVFs within thin chambers, where the temperature field shows minimal variation perpendicular to the plates and theoretical work demonstrates that fluid flow in that direction is negligible^51,53^. These conventional approaches, typically implemented at relatively low scanning rates (few kHz), often rely on a single, repetitive 1D scanning path^46,47^ (Fig. 1b).

The first design novelty of this work is the implementation of high-frequency line-by-line scanning scheme (Fig. 1c) within a thicker chamber, optimized through a carefully tuned ratio between the *x*- and *y*-ranges, together with precise control of the overall scan repetition rate. In thicker chambers, the 2D assumption does not hold anymore, and physical considerations dictate higher temperatures in the scanning plane where the focus is narrower and hence, the thermoviscous transport is stronger. As a direct consequence, this strong localization to the scanning plane induces the out-of-plane rotational flows not observed in thin chambers. Our approach allows flexible tuning of the coupling between in-plane and out-of-plane TVFs, thereby achieving precise 3D helical particle manipulation. Notably, our measurements indicate that at a laser power of 25 mW, the induced flow is readily observable, accompanied by an average temperature increase of about 2 K (Fig. S1). The precise design of the scan pattern, specifically the temporal ordering of the scan, can also profoundly influence the resulting flow fields (Fig. S2), offering additional degrees of freedom beyond the classic 1D scanning, as we return to later on.

Moreover, as shown in Fig. 1d, the vertical position of the laser scanning plane within the chamber plays a crucial role in practice. When the laser is focused at the center of the chamber, symmetrically positioned between two identical walls, we observe predominantly 2D TVFs within the focal plane. This does not imply the absence of 3D flow components, but rather that any out-of-plane components are beyond the imaging plane and thus are not visible. In sharp contrast, if the scanning plane is positioned near the top surface, strong 3D helical TVFs, with a pronounced *z*-component, become observable. This asymmetric configuration provides a practical strategy for generating helical flows with controllable handedness throughout the vertical depth of the chamber.

We next sought to characterize the flow field at single-particle resolution. To this end, we applied a 3D stop-flow microscopy protocol. As illustrated in Fig. 1e, this experimental pipeline allows asynchronous manipulation and tracking of individual particles by combining the induced TVFs with a confocal microscope’s sectioning capability to localize particles in 3D. This method leverages a key advantage of our system: the use of a highly viscous medium (18.8 Pa·s at 25 °C). In such an environment, Brownian motion is strongly suppressed, and both gravitational and inertial forces are negligible. Consequently, particles remain nearly stationary when the laser is turned off, enabling acquisition of confocal *z*-stacks comprising tens to hundreds of images to accurately determine vertical positions. The measured 3D trajectory of a single particle not only confirms the expected helical structure of the induced flow field, but also reveals a damping behavior that emerges over time (Fig. 1f). This phenomenon offers a fully new approach to opto-hydrodynamic focusing of particles, which previously required the use of sheath fluid streams to achieve^44,54^.

### Formation, tunability, and theoretical understanding of 3D helical TVFs

**Fig. 2:**
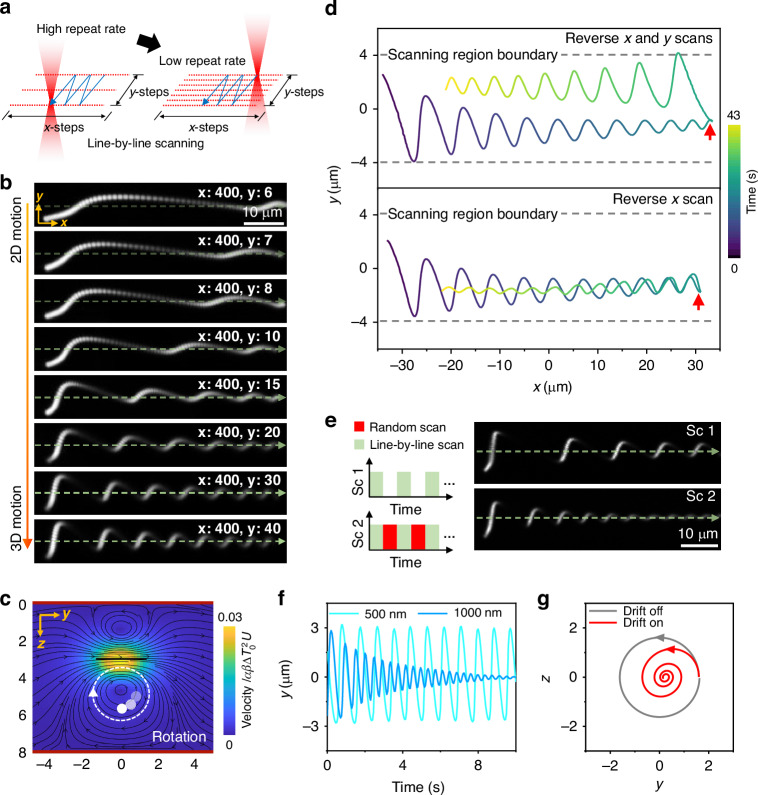
Tunable 3D helical TVFs and opto-hydrodynamic focusing of single particles. **a** Schematic of a line-by-line laser scanning trajectory in the *x*–*y* plane with a tunable repetition rate. The maximum scanning ranges along both *x* and *y* are fixed, making the repetition rate dependent on the step sizes in the *x*- and *y*-directions. **b** Maximum intensity projections of a single fluorescent particle driven by the TVFs over time, revealing the transition from dominant 2D in-plane transport to pronounced 3D helical rotation with focusing. The dashed line in each panel denotes the principal rotation axis. The *x*-direction scan step is fixed at 400 steps, while the *y*-direction scan steps are varied from 6 to 40, thereby modulating the coupling strength between the out-of-plane and in-plane components of the TVFs. The laser power used in these experiments was fixed at 25 mW. **c** Minimal model of 3D TVFs and theoretical average velocity of tracer particles in viscous fluid flow confined between no-slip parallel plates (*z* = 0, 8), driven by a heat spot scanning along a path (length 2.75) in the negative *y*-direction in the plane *z* = 3. Here, lengths are normalized by the characteristic heat-spot radius and the velocity is normalized with respect to its characteristic scale, $\alpha \beta \Delta {T}_{0}^{2}U$, where $\alpha$ is the thermal expansion coefficient, $\beta$ is the thermal viscosity coefficient, $\Delta {T}_{0}$ is the peak instantaneous temperature increase and $U$ is the speed of laser translation^53^. Out-of-plane components from the translating, axially modulated, and asymmetrically positioned temperature field generate helical flows with broken symmetry. **d** Driving of a single microparticle with reversal of laser scan direction, highlighting the vectorial character of TVF actuation. Red arrows mark the particle’s position at the moment of scan reversal: both *x*- and *y*-directions reversed (top panel) and only the *x*-direction reversed (bottom panel). **e** Effects of temporal delay and random heating on particle focusing. **f** Experimental demonstration of particle size-dependent opto-hydrodynamic focusing. **g** Simulated trajectories of a single particle with and without a unidirectional, *z*-dependent drift. The particle is initiall*y* placed at position (*y* = 1.6, *z* = 0)

To reveal the formation, tunability, and physical mechanisms of 3D helical TVFs, we vary the number of scanning steps along the *y*-direction, while keeping both the number of *x*-direction steps and the scanning ranges in both directions constant (Fig. 2a). This approach effectively modulates the repetition rate of the entire scanning cycle. Figure 2b presents the maximum intensity projections over time of a single fluorescent probe particle driven by the TVFs under different scanning conditions. In these experiments, a reduction in fluorescence intensity was interpreted as the particles moving out of the imaging plane. A pronounced transition is observed from 2D in-plane transport to fully developed 3D helical rotation, accompanied by robust focusing (see also Video S1). Moreover, the strength of the helical TVFs is sensitive to the temperature-dependent viscosity of the medium. Stronger variation of viscosity with temperature could lead to a larger helical flow velocity (Fig. S3).

We propose that helical flows result from a combination of generalized planar motion in the *y*-*z* plane, such as rotational motion, and concurrent linear translation along the *x*-axis. The resulting particle trajectory traces out a helical path in 3D space.

In our system, the short-range, high-frequency, *y*-directional scanning induces a strong out-of-plane circular flow, which couples with orthogonal flow components generated by the effective long-range, lower frequency, *x*-direction scanning, thus giving rise to the 3D helical structure. This view is further supported by theoretical modeling (Fig. 2c), which confirms that an axial modulation of a linearly translated heat profile induces vortices with out-of-scanning-plane flow components that become particularly pronounced when the laser scanning is performed with an axial offset to one side of the chamber. It is noteworthy that these out-of-plane flow profiles closely resemble the dipolar flow fields that are typically observed in-plane^2,46^ (see Methods section for theoretical explanation of 3D TVFs and range of validity of TVF model). These simulated profiles are consistent with our experimental measurements of *z*-position dynamics. By tracking the particle’s fluorescence intensity as a proxy for its *z*-position dynamics (Fig. S4), we find that its velocity decreases when it is farther away from the scanning plane, where it spends more time, compared to when it is closer. As the focusing stabilizes, the fluorescence intensity also reaches a steady state. This steady-state intensity is lower than the maximum fluorescence intensity, suggesting that the particle’s steady-state *z*-position lies below the scanning plane.

We further check the correlation between laser scanning directions and the particle’s helical rotation dynamics within the scanning region. Figure 2d shows the trajectories of a single particle (dashed lines denote the scanning region boundaries) when both *x*- and *y*-scan directions are reversed simultaneously (red arrow). In this case (top panel, also see Video S2), reversing the *y*-scan direction flips the handedness of the helical flow, while reversing the *x*-scan direction inverts its propagation direction, a direct signature of the thermoviscous actuation.

The particle’s average *y*-position is displaced from the *y*-center of the scanning region, showing a stronger preference for one boundary, either +*y* or −*y*. To elucidate this effect more explicitly, we employed a single repeated line scan, which produced an asymmetric flow profile along the ±*y* direction (Fig. S5). Specifically, when the scan was performed along the positive *y*-direction, the flow center shifted toward +*y*, whereas reversing the scan direction resulted in a shift of the flow center toward –*y*. The resulting total flow profile becomes asymmetric, with its center shifting depending on the scanning direction^49^; this could in turn provide the systematic offset of the helical flow axis in the ±*y* direction seen in Fig. 2d. Such asymmetry becomes clearly observable in experiments when the scanning speed is relatively slow (Fig. S5) and could originate from asymmetry of the temperature profile.

Notably, reversal of the *x*-scan direction indicates translational invariance of the physical driving mechanism of the rotation around the *x*-direction (bottom panel of Fig. 2d, also see Video S3), which we will later use to enhance particle focusing within a limited scanning distance.

### Opto-hydrodynamic focusing of single particles

To shed light on the underlying mechanism of this opto-hydrodynamic focusing effect and to identify the key parameters that govern its tunability, we performed a series of systematic experiments. In power-dependent TVF actuation experiments, both the particle trajectories and the focusing height remain essentially unchanged over the explored laser power range (Figs. S6–7), suggesting that the focusing behavior is not governed by the flow strength. Furthermore, we introduced randomized scanning within the same spatial region (Fig. 2e). This strategy enables a more direct evaluation of thermal effects on particle focusing without altering the laser power or scan range. As shown in Fig. 2e, the focusing effect is markedly enhanced. Note that randomized scanning alone is insufficient to achieve particle stabilization along either the *y*- or *z*-directions. Moreover, we find that the focusing of particles can be finely controlled by particle size (Fig. 2f and Video S4). Small particles do not converge efficiently, whereas larger particles experience stronger damping and form stable spiral trajectories (Fig. S8).

Based on these observations, we developed a phenomenological description comprising a rotational flow field in the *y*-*z* plane, combined with an additional velocity contribution, modelling particle-size-dependent thermophoretic, hydrodynamic or other physical effects, acting along the *z*-direction and described by a low-order polynomial in *z* (see “Methods” and Fig. S9).

Remarkably, numerical simulations reveal that a unidirectional, *z*-dependent drift term acting on the particle, without requiring any sign reversal, can effectively switch the focusing behavior (Fig. 2g). The experimentally observed focusing can therefore be understood as a consequence of the coupling between the rotational flow field and a drift term.

A similar size-dependent behavior is observed even under simple one-dimensional laser scanning, where particles of different sizes exhibit consistently damped motion in the *x*–*y* plane (Fig. S10). Together, these results demonstrate the efficiency of TVFs for tunable out-of-plane rotation in highly viscous media and introduce a new dimension of control for optofluidic systems.

### Selective isolation of 3D rotational components via symmetrized scan paths enables stable out-of-plane rotation and spin control

**Fig. 3:**
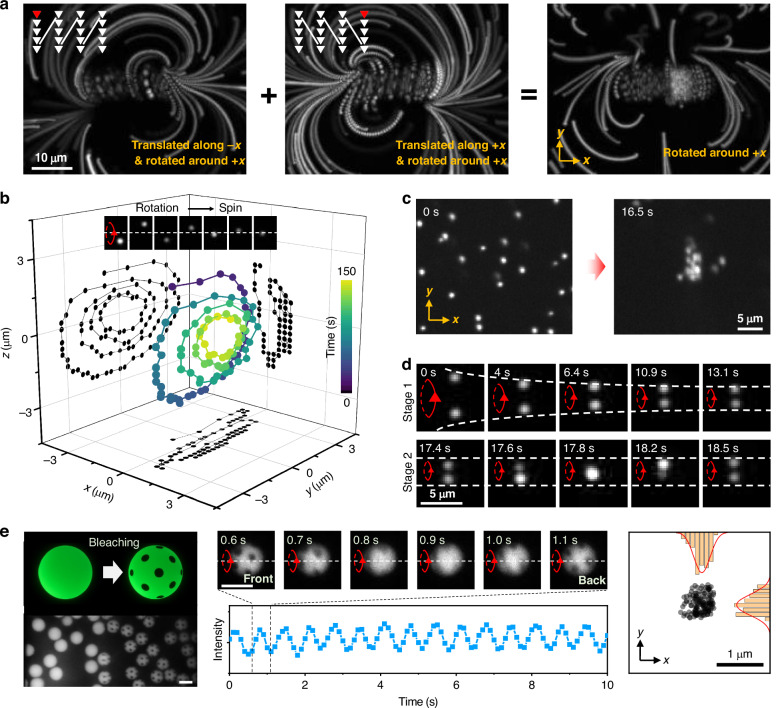
3D helical TVFs enable stable out-of-plane rotation and stationary spinning of microparticles in high-viscosity environments. **a** Rational design of rotation-dominated flow fields. Left: 3D helical TVFs induced by scanning in the positive *x*-direction propagate along the negative *x*-direction. Middle: 3D helical TVFs propagate to the positive *x*-direction by scanning in the negative *x*-direction. Right: A back-and-forth line-by-line scanning sequence largely eliminates the net flow along the *x*-direction. **b** Measured 3D single-particle trajectory reveals opto-hydrodynamic rotation and focusing of the particle (with a diameter of 1 μm) at a stable *x*-position. **c** Demonstration of particle accumulation with high throughput, where dense clustering occurs within only a few seconds of illumination. **d** Evolutionary pathway from rotation to binding, leading to a stable spinning dimer. **e** Left: Schematic (top) and experimental image of the photobleaching of individual fluorescent, perfectly spherical microparticles. Middle: Measured fluorescence intensity of a stably spinning, inhomogeneously photobleached particle as a function of time. Right: Histogram of the particle’s *x* and *y* positions. The particle trajectory is tracked using ImageJ to extract time-resolved *x*- and *y*-coordinates, whose distributions are fitted separately with Gaussian functions. The resulting ${\sigma }{x}=167\,{\rm{nm}}$ and ${\sigma }{y}=186\,{\rm{nm}}$ characterize the particle’s positional fluctuations. The scale bars are 10 μm

We next investigate whether confinement can also be achieved along the *x*-axis, thereby fully restricting the particle in all three spatial dimensions and allowing only rotational/spinning motion. Following a rational design route, motivated by the symmetry classes observed above, we intentionally symmetrize the scan pattern further by alternating between scan patterns with positive and negative *x*-scanning, which effectively cancels the in-plane TVFs along *x*-axis while preserving the out-of-plane vortex component (Fig. 3a). As shown in Fig. 3b, the measured 3D trajectory of a single fluorescent probe particle exhibits strong confinement along *x*, with a distinct spiral motion in the *y*-*z* plane. This approach also proves effective for multiple particles: they are drawn toward the central region and rotated out-of-plane (Fig. 3c, see also Video S5). By employing more sophisticated temporal control, we can induce pure out-of-plane rotations with opposite directions to occur simultaneously (Fig. S11). In this case, the rotations are confined near the scanning region boundary, in correspondence with the top panel of Fig. 2d.

As particles interact with the flow, opto-hydrodynamic focusing gradually stabilizes their motion, driving them into a steady spinning state. To examine this process, we tracked two particles (1 μm-diameter) simultaneously engaged by the TVFs. Their interparticle distance steadily decreases as they co-rotate (stage 1, Fig. 3d), ultimately forming a rigid, spinning dimer (stage 2, Fig. 3d, also see Video S6). Interestingly, when larger particles are used, the out-of-plane rotation can be suppressed, and the particles directly enter a stable spinning state. To verify that spinning is not an artifact from residual optical forces, we employed a perfectly spherical fluorescent microsphere (10 μm-diameter), inhomogeneously labeled via localized in-situ photobleaching (Fig. 3e, also see Video S7). Time-resolved fluorescence monitoring revealed periodic variations, confirming that stable spinning of particles can be reliably achieved. Additionally, position tracking in the *x*–*y* plane further verified stable opto-hydrodynamic trapping throughout the spinning process (Fig. 3e). These results emphasize that no special geometry of the particle is required for rotation; even perfectly round particles are compatible. This presents an advantage for practical applications in which the shape of particles often cannot be controlled.

Moreover, we have conducted systematic measurements using polystyrene spheres of two different diameters under varying scan widths. The results reveal a clear dependence of positional stability on both particle size and scanning parameters: larger particles and narrower scan widths exhibit reduced positional fluctuations, whereas smaller particles or wider scan widths result in increased positional variance (Fig. S12). Specifically, at a fixed scan repetition rate, increasing the *y*-scan range could lead to a pronounced deterioration of the focusing quality for particles with a diameter of 1 µm (Fig. S13).

Importantly, the rotational motion can be discretized (i.e., “stop-and-go” rotational control). By introducing a temporal delay in the scanning sequence, we achieved stepwise rotational control analogous to a stepper motor (Fig. S14, see also Video S8). This level of tunability is made possible by the highly viscous medium (18.8 Pa·s at 25 °C), along with the kinematic nature of TVFs: defining features of our approach. Under near-physiological conditions, for which the viscosity is substantially lower, the TVF actuation mechanism still works, but with diminished positional precision. In terms of the deterministic theory for TVFs, the flow velocity is independent of absolute viscosity of the fluid. However, thermal noise of course affects the motion of tracers in experiments. Using a scaling argument, we may obtain a theoretical estimate for the threshold viscosity, above which flow is dominant. First, we may estimate the diffusivity $D$ for a tracer bead of radius $b$ from the Stokes–Einstein relationship as $D\approx {k}_{B}{T}_{0}/6\pi {\eta }_{0}b$, where ${k}_{B}$ is Boltzmann’s constant, ${T}_{0}$ is the reference absolute temperature, and ${\eta }_{0}$ is the reference viscosity of the fluid. Then the Péclet number, quantifying the effect of advection versus diffusion, is given by ${\rm{Pe}}\sim {Vb}/D \sim \,6{\rm{\pi }}{{\rm{\eta }}}_{0}{b}^{2}V/{k}_{B}{T}_{0}$, where *V* is the flow velocity. Noise becomes comparable with advection when Pe is on the order of 1 (whereas a large Péclet number corresponds to flow dominating noise), so that the threshold viscosity is given by ${{\rm{\eta }}}_{0}\sim {k}_{B}{T}_{0}/6{\rm{\pi }}{b}^{2}V$. Note that larger particles are less impacted by noise, because the theoretical threshold viscosity scales as ${\eta }_{0}\sim 1/{b}^{2}$. Given that ${k}_{B}=1.38\times {10}^{-23}\,{{\rm{m}}}^{2}$ kg ${{\rm{s}}}^{-2}\,{{\rm{K}}}^{-1}$, then with ${T}_{0}=298$ K, *b* = 0.5 μm (for example) and $V\approx 3$ μm/s, we obtain ${{\rm{\eta }}}_{0}\approx$ 0.3 mPa·s.

An alternative, more experimentally oriented viewpoint would be to define the characteristic length scale of the system as the diameter of the helical flow fields, denoted by *L*. This would replace the scaling ${\eta }_{0}\sim 1/{b}^{2}$ with ${\eta }_{0}\sim 1/{bL}$. In practice, *L* is usually around one order of magnitude larger than the particle size *b*, which in turn leads to a lower estimate of the critical viscosity.

These theoretical estimates are smaller than the threshold viscosity found in the experiments, which is in the range of 30–65 mPa·s (at 25 °C). However, our calculation assumed that the TVF speed is approximately the same in the different viscosity media. In contrast with this, at low viscosities, the TVF speed in experiments was also suppressed. Diffusion could therefore dominate advection by flow at higher viscosity than predicted by the scaling argument. Furthermore, the effects of optical forces and thermophoresis may also compete with the TVFs at lower viscosities.

### 3D helical TVF-based multi-view microscopy for high-resolution imaging

**Fig. 4:**
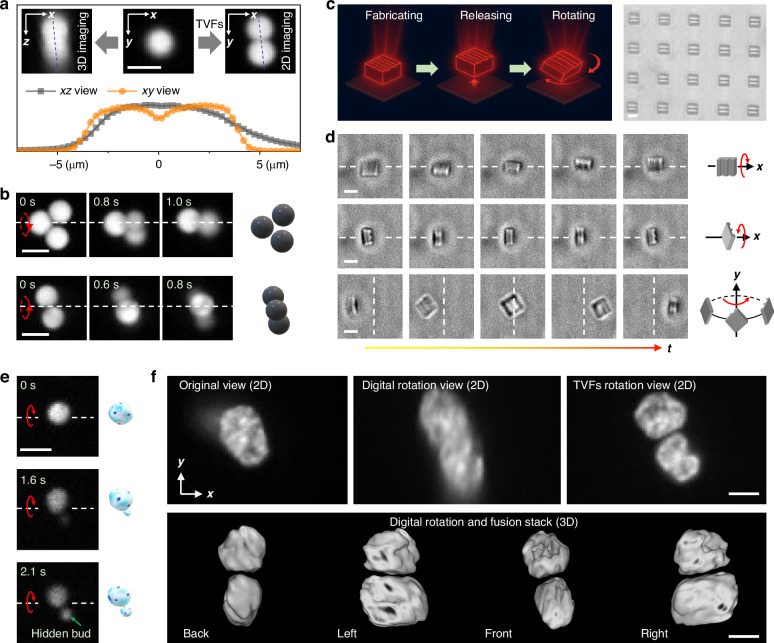
Out-of-plane rotations for improved imaging of suspended assemblies, nano-manufactured particles, and biological cells. **a** TVF-driven reorientation of a dimer structure to align its original axis of symmetry with the *x*–*y* imaging plane, significantly enhancing the visibility of the interparticle gap and highlighting advantages over conventional 3D confocal microscopy in resolving specific features. **b** Orientation-resolved 2D imaging of a self-assembled trimer around different rotational axes. **c** Schematic overview of the controlled release and rotation of substrate-anchored microstructures fabricated via two-photon lithography, illustrating the robustness and flexibility of the helical TVFs. **d** Out-of-plane rotation of a single micro-tile about different rotational axes, whose surfaces exhibit engraved patterns. **e** Out-of-plane rotation of a yeast cell, enabling visualization of the hidden bud. **f** Multi-view microscopy for high-resolution 3D imaging of HCT116 cells. While an original image stack did not resolve two cell nuclei even after digital rotation into the *x*–*y* plane, TVF rotation into plane allows the gap between the nuclei to be clearly resolved. After multi-view image fusion (lower row), this gap can be observed from any axial orientation. The scale bars are 5 μm

Multi-view strategies are widely recognized as an effective means to overcome the intrinsic anisotropy of 3D optical microscopy. Figure 4a illustrates a scenario in which critical structural features, such as the interparticle gap in a dimer, are aligned along the optical (*z*) axis and are therefore difficult to resolve using standard *z*-sectioning, but become more readily accessible when the dimer is rotated into the *x*–*y* plane. However, conventional implementations typically rely on multiple objectives or specialized sample-mounting geometries^55^, which limit access to densely sampled viewing angles and frequently constrain the accuracy of 3D imaging. Although microrotation platforms offer a potential route^56^, they are limited to a single axis, and integrating multi-view strategies with such systems remains complex and lacks reconfigurability. Optical-tweezer-based approaches also face particular challenges in simultaneously achieving stable *z*-axis trapping and controlled out-of-plane rotation^32^.

In our approach, rotation is achieved through the helical TVFs rather than optical torque, while the use of a high-viscosity medium effectively suppresses Brownian fluctuations and passively stabilizes the particle along the *z*-axis when the laser is switched off. More importantly, the rotation driven by TVFs is usually around the object center (spinning), which is not usually the case with mechanical manipulators without dedicated compensation mechanisms^56,57^.

To highlight the advantages of our approach over conventional micromanipulation techniques for multi-view microscopy, we present several proof-of-concept experiments. First, we show the capability of TVFs becomes especially powerful for complex assemblies (also see Fig. S15), such as trimers consisting of three particles of diameter 4 μm (Fig. 4b, also see Video S9), where multiaxial rotation is also feasible. Note that recent photothermal strategies for out-of-plane rotation rely on localized forces and are thus restricted to individual particles^17^, failing in assembled structures where particles are only loosely bound. By contrast, our approach leverages 3D helical TVFs acting collectively, enabling controlled rotation of both single particles and assemblies.

Second, we show that our TVFs are more robust for artificial structures that are typically difficult to rotate. Thin micro-tiles, for example, pose a challenge due to their high aspect ratio. Using micro-tile arrays fabricated via two-photon polymerization (Fig. 4c), we demonstrate that the combination of laser-induced heating and TVFs can peel a single micro-tile from the substrate (Video S10). Unlike the aggregation observed in Fig. 3c, this process isolates and detaches an individual tile, revealing a distinct manipulation capacity. Once released, these TVFs enable robust multimodal rotational control of the micro-tiles (Fig. 4d, also see Video S11).

Finally, we demonstrate the applicability of our method to cells, which highlights the versatility of our approach in bioimaging. In Fig. 4e, we show the out-of-plane rotation of a single yeast cell, which reveals a hidden bud that was initially located outside the imaging plane (see also Video S12). As a more comprehensive demonstration, we show how TVFs enable multi-view microscopy for high-resolution 3D imaging of HCT116 cells. In Fig. 4f (top panel), the stop-and-go capability for stepwise in-place spinning allows repeated volumetric imaging of the same sample from different orientations, thereby implementing a practical multi-view 3D workflow. From the original dataset, in which only one apparent cell nucleus is observed, digital rotation does not allow us to identify and further segment the individual cells forming the cluster. In contrast, after rotation driven by TVFs, two different delimited cell nuclei are clearly observed. Subsequent fusion of the multi-view datasets yields voxel-based 3D reconstructions, without the resolution limitations along the *z*-axis that are typical of single-view confocal imaging (bottom panel of Fig. 4f). Helical TVF fusion can therefore effectively enhance the resolution of 3D microscopy while leveraging established image fusion algorithms.

In the case of yeast cells, no major changes in morphology were observed after embedding them in the chamber. Considering that yeasts are known to survive in highly viscous media^58^ and that the exposure time for each sample was always shorter than 1 h, this outcome was expected. For mammalian cell lines, samples were fixed with paraformaldehyde (PFA) to prevent structural damage induced by the medium conditions. Since the cells were maintained in suspension, thereby reducing the structural implication of the cytoplasm, no clear signs of nuclear damage were observed within the experimental time window (up to ~30 min per sample). In addition, we performed experiments using live cells in the same viscous medium. Although cell swelling was observed in some samples, particularly after prolonged observation times (>30 min), nuclear structures retained morphological features similar to viable cells.

We acknowledge that normal physiological conditions and viability of live cells could be compromised in this medium. Consequently, the use of fixative agents is required, which is a common practice in sample preparation for many microscopy techniques. The compatibility of the viscous medium required for TVFs operation with live mammalian samples will be investigated in future work.

## Discussion

By scanning a single laser across a 2D plane, we synthesize fully 3D helical TVFs and tune their structure via scan geometry and timing, thereby adding novel and robust out-of-plane degrees of freedom to TVF-based micromanipulation. A key insight is that the balance of in-plane and out-of-plane flow components can be robustly tuned by adjusting laser scanning parameters such as scan length, directionality, and repetition rate. Our approach can be further extended to generate transverse helical TVFs that propagate not only along straight centerlines, but also along arbitrarily curved trajectories (Fig. S16 and Video S13), paving the way for yet more advanced 3D manipulations. Here, we have discovered tunable opto-hydrodynamic focusing of microparticles with 3D TVFs, which can be interpreted as an emergent consequence of the coupling between the rotational flow field and a drift term. Notably, such focusing itself is a long-standing challenge in microfluidics, typically requiring complex solutions such as sheath flows and hierarchical channel designs^45^. Our method could potentially facilitate the rational design of particle-sorting flow fields.

In the present work, we also incorporate the convergence of orbital trajectories into novel microscopy techniques. By exploiting symmetry in bidirectional scan patterns, we decouple rotation from lateral drift and achieve stable spinning with sub-200 nm positional fluctuations across perfectly spherical beads, nano-printed high-aspect ratio tiles, and stained biological cells.

As concrete applications, we demonstrate utility in microscopy, where cells can either be rotated through the optical plane for live inspection or more systematically imaged from multiple angles to facilitate improved resolution via multi-view fusion strategies.

Despite continued advances in the field, high-viscosity media that suppress diffusion pose significant difficulties for existing micromanipulation platforms attempting to translate, accelerate, confine, or rotate particles via applied forces or optofluidic approaches^25^. Here, we turn this apparent limitation of high viscosity into an advantage, enabling higher precision, kinematic stop-and-go actuation of arbitrary micro-objects. Taken together, the presented advances establish a new paradigm in microscale flow control for micromanipulation in the life and engineering sciences, and constitute a significant step toward a future of generally applicable 3D microrobotics.

## Materials and methods

### Instrumentation and materials

The experimental setup integrates a custom-built infrared laser scanning system (1455 nm, CRFL-20-1455-OM1, Keopsys) with an inverted microscope (IX83, Olympus) for both laser projection and fluorescence imaging. A relay lens configuration (4 f system) reimages the output plane of the acousto-optic deflector (AOD; AA.DTSXY-A6-145, AA Optoelectronics) onto the back aperture of the objective lens (UPLSAPO60XW, NA 1.2, Olympus), enabling precise beam steering at the sample plane. The microscope is equipped for bright-field and fluorescence imaging and incorporates a spinning disk confocal unit (CSU-X1, Yokogawa) for high-resolution imaging with reduced background and accurate z-sectioning of both fluorescent and non-fluorescent specimens. The AOD is driven by a custom LabVIEW program via a PCI Express data acquisition card (PCIe-6363, National Instruments). The laser heating and optical manipulation are achieved using the 1455 nm laser, whereas the confocal fluorescence imaging is carried out with a separate 488 nm excitation laser. Fluorescent polystyrene (PS) microparticles were purchased from Polysciences Europe GmbH. A high-viscosity medium (18.8 Pa·s at 25 °C) composed of 40% fructose, 30% glucose, 10% sucrose, and 20% water by weight was used to embed PS beads or two-photon printed microstructures in the sample chamber.

### Preparation of biological samples

The HCT116 cells (ATCC, CCL-247) were cultured in DMEM + GlutaMAX (Gibco) supplemented with 10% FBS (Gibco) and 1% penicillin-streptomycin (Gibco) at 37 °C and 5% CO_2_. Cells were tested as negative for mycoplasma infection. Prior to exposure to TVFs, cells were stained with NucBlue Live ReadyProbes Reagent (Invitrogen) for 20 min and then fixed with 4% PFA in DPBS (Gibco) for 10 min. Afterwards, cells were washed twice with DPBS and resuspended in DI Water. Cell suspension was mixed with the high-viscosity medium in a 10:90 proportion prior to being embedded in the sample chamber. For budding yeast rotation, a *Saccharomyces cerevisiae* (*S. cerevisiae*) strain, with W303 background (yJHK112) originally prepared with ymCherry constitutively expressed under an ACT1 promoter to induce fluorescent labeling, was used. The strain was created by Andrew Murray’s lab and kindly gifted by Jona Kayser^59^. Yeasts were cultured in YPD agar (Sigma Aldrich). To create the sample chamber, colonies were picked, resuspended in DI Water and mixed with the high-viscosity medium in a 10:90 proportion.

### Two-photon lithography

The micro-tile arrays were fabricated via 3D laser microprinting based on two-photon-absorption (Professional GT, Nanoscribe)^60^. Herein, a femtosecond laser at 780 nm center wavelength was focused through a 63×, 1.4 NA objective lens (Carl Zeiss) and scanned through a droplet of liquid photoresist (IP-Dip) at a focus velocity of 0.01 m s^−1^ to create the structures. After printing, the samples were developed for 10 minutes in 1-methoxy-2-propanyl acetate (PGMEA), followed by 10 minutes in isopropyl alcohol and gently blown dry with nitrogen gas. In order to facilitate damage-free release, the structures were printed on a sacrificial layer with a thickness of around 150 nm. This was achieved by spin-coating (KL-SCE-150, Quantum Design GmbH) a solution of 7% (w/w) polyvinyl alcohol (PVA) in water onto the substrate (ITO-coated glass, 30 × 30 × 0.7 mm, Nanoscribe) with 67 rps for 60 s and subsequently baking on a hotplate at 80 °C for 2 min.

### Theoretical explanation of 3D TVFs

First, in the idealized mathematical (i.e., asymptotic) limit where the temperature variation is spherically symmetric and the confining geometry is absent (i.e., in 3D unbounded space), the flow behavior associated with a heat spot scanning along a line segment is axisymmetric about this scanning axis, with strong net thermoviscous flow near the scan path and recirculation further away^61^. The experimentally relevant geometry (i.e., a microfluidic chamber that is thick relative to the characteristic heat-spot radius) may be treated as a perturbation of this fully 3D, unbounded space. The introduction of chamber walls, on which the fluid flow obeys no-slip boundary conditions, modifies the flow induced by a heat spot in unbounded space. Mathematically, we assume that the heat spot is far from the chamber walls (relative to its radius). We approximate the flow far from the heat spot using classical hydrodynamic singularities of incompressible inertialess flow (known as Stokes flow^62^); these viscous flow singularities are analogous to solutions for a point charge and point dipole in electrostatics. We may then find the perturbation to the fluid flow in unbounded space, induced by the presence of the no-slip walls, in terms of hydrodynamic image singularities, by an iterative process known as the method of reflections^53^. Averaging the flow over a scan period finally yields the net thermoviscous transport of passive tracers (Fig. 2c). In contrast with the unbounded case, the resulting vortices have finite size, due to confinement between the chamber walls.

### Range of validity of TVF model

Within the theoretical model, although we cannot place the laser scanning plane exactly at the chamber wall to replicate the experimental geometry, we may place it three characteristic heat-spot radii away (i.e., sufficiently far for model validity) from the nearest chamber wall. Then, for illustrative purposes, we set the overall chamber height as eight times the characteristic heat-spot radius. This allows us to demonstrate the formation of a larger vortex between the laser scanning plane and the further chamber wall, providing theoretical justification for the asymmetric positioning of the laser scanning plane within the chamber in experiments. Then we may set the heat-spot radius (4 µm), length of the scan path, and the scan frequency according to the experimental values.

First, we consider the experimental scan pattern for helical TVFs as consisting of many short scan paths. To gain physical understanding of the rotation component of the helical motion, we consider one short scan path in the −*y* direction. By integrating the average velocity field of tracers, we may find the trajectory of a tracer particle.

With the theoretical model, considering one short scan path in the −*y* direction (length 7.6 µm, scan frequency 500/50 = 10 kHz), we may then find the rotation period, i.e., the time taken for a particle to complete a closed trajectory. A typical experimental value for this is around 2 seconds (Fig. 2d, top panel). Our asymptotic model can also produce rotation periods of similar magnitude, for example, around 10 s for a particle starting 1.6 times the characteristic heat-spot radius below the laser scanning plane. This uses peak instantaneous temperature change Δ*T*_0_ = 8 K, constant heat-spot amplitude, dimensionless thermal expansion coefficient *α* = 0.004, and dimensionless thermal viscosity coefficient *β* = 0.6, with *α* estimated from the literature^63^ and *β* from our measurements.

Next, the translation component of the helical trajectories arises from the progression of the laser along the scan pattern in the −*x* direction. To estimate the pitch of the helix, we may think of the scan pattern instead as made up of long scan paths in the −*x* direction. We consider one long scan path (length 68 µm) in the −*x* direction and see how far a particle translates in the *x* direction over the course of a rotation period estimated above. This can give a helix pitch of the experimentally observed order of magnitude, 3 µm.

### Phenomenological model of opto-hydrodynamic focusing

We consider the overdamped dynamics of a point-like particle confined to the *y*-*z* plane and immersed in a simplified prescribed background flow field. The particle is additionally subject to an external force acting along the *z*-direction. Inertial effects are neglected, which is appropriate for micron-sized particles in a viscous fluid flow at low Reynolds number.

The background flow field is modeled as a two-dimensional rotational flow with a constant angular velocity ${\omega }_{0}$, representing the minimal rotational field required to generate sustained azimuthal motion. The velocity field is given by ${\bf{u}}\left(y,z\right)={\omega }_{0\,}(-z,y)$; this term is divergence-free and by itself would generate closed circular streamlines centered at the origin.

Under overdamped conditions, the particle velocity is determined by the superposition of the local flow velocity and the drift induced by external forces. The equation of motion reads

$$\frac{d}{{dt}}\left(\begin{array}{c}y\\ z\end{array}\right)={\bf{u}}\left(y,z\right)+\left(\begin{array}{c}0\\ -\mu F(z)\end{array}\right)$$

where *μ* denotes the particle mobility. The external force is assumed to depend only on the *z*-coordinate and is approximated by a linear form $F\left(z\right)=S({a}_{0}+{a}_{1\,}z)$.

### Multi-view fusion

The multi-view fusion is implemented as a two-step process, consisting of a 3D alignment step and a 3D fusion step. The input data are an arbitrary number $n$ of volume images of the sample, ${I}_{1},\ldots ,{I}_{n}$, recorded with the stepwise stop-and-go capability of the proposed technique. The sample orientations (respectively, view directions) of the volume images need not be known a priori. In addition to the image data, the 3D point spread function (PSF) is also required.

The alignment step estimates the sample orientations. It is implemented in a custom MATLAB script (MATLAB R2025a, MathWorks, Natick, MA, USA). The input volume images are first deconvolved with Lucy-Richardson deconvolution^64^, considering the given PSF. The deconvolved volumes are then aligned using rigid, mono-modal (intensity-based) image registration, using the first volume as the fixed reference. The result data of the alignment step are $n-1$ rigid 3D transformation matrices, ${T}_{21},\ldots ,{T}_{n1}$, that describe the estimated 3D rotation and translation required to align ${I}_{2},\ldots ,{I}_{n}$ with the reference ${I}_{1}$.

The fusion step montages the aligned volume images. It uses the open-source BigStitcher plugin^65^ for Fiji^66^. After creating a multi-view dataset that contains the $n$ original volume images, the alignment information is applied to ${I}_{2},\ldots ,{I}_{n}$ by explicit specification of their respective transformation matrices transformations ${T}_{21},\ldots ,{T}_{n1}$, and the PSF is assigned to every view. Finally, multi-view deconvolution^67^ is performed to obtain a single, fused volume representation of the sample without the spatial resolution limitations along the *z*-axis. Multi dimension views of cells after multi-view fusion reconstruction were obtained using napari software.

## Supplementary information

Supplementary Materials

Video S1

Video S2

Video S3

Video S4

Video S5

Video S6

Video S7

Video S8

Video S9

Video S10

Video S11

Video S12

Video S13

## Data Availability

All relevant data supporting the findings of this study are available from the corresponding author on request.
