# Supplementary material for: Helical opto-thermoviscous flows drive out-of-plane rotation and particle spinning in a highly viscous micro-environment
Source: Light Sci Appl. 2026 May 11;15:231. doi: 10.1038/s41377-026-02303-8 (PMC13161375; doi:10.1038/s41377-026-02303-8)
Supplement: Supplementary file 1 — Supplementary Materials [file 41377_2026_2303_MOESM1_ESM.pdf]

Supplementary Materials for

Helical opto-thermoviscous flows drive out-of-plane rotation and particle spinning in a highly viscous micro-environment

Fan Nan[1]*, Weida Liao[2†], Adrián Puerta[1], Josephine Spiegelberg[3], Elena Erben[1], Ralf Mikut[4], Stephan Allgeier[4], Martin Wegener[3,5], Eric Lauga[2] and Moritz Kreysing[1]*

[1]Institute of Biological and Chemical Systems, Karlsruhe Institute of Technology, Eggenstein-76344 Leopoldshafen, Germany

[2]Department of Applied Mathematics and Theoretical Physics, University of Cambridge, Cambridge CB3 0WA, UK

[3]Institute of Nanotechnology, Karlsruhe Institute of Technology, 76344 Eggenstein-Leopoldshafen, Germany

[4]Institute for Automation and Applied Informatics, Karlsruhe Institute of Technology, 76344 Eggenstein-Leopoldshafen, Germany

[5]Institute of Applied Physics, Karlsruhe Institute of Technology, Wolfgang Gaede-Straße 1, 76131 Karlsruhe, Germany

[†]Present address: Department of Mathematics, Imperial College London, London SW7 2AZ, UK

*e-mail: fan.nan@kit.edu and moritz.kreysing@kit.edu

Supplementary Videos

Video S1: Single fluorescent particles (1 μm in diameter) driven by helical TVFs under different scanning conditions.

Video S2: A single fluorescent particle (1 μm in diameter) driven by programmable TVFs at the moment when both the *x*- and *y*-scan directions of the laser are reversed simultaneously.

Video S3: A single fluorescent particle (1 μm in diameter) driven by programmable TVFs at the moment when only the *x*-scan direction of the laser is reversed.

Video S4: Out-of-plane rotation of fluorescent particles (1 μm and 0.5 μm in diameter). For the larger particle, hydrodynamic focusing occurs, leading its spiral motion to converge toward a stable particle height.

Video S5: Out-of-plane particle rotation and accumulation of multiple fluorescent polystyrene particles (0.5 μm in diameter).

Video S6: Out-of-plane rotation and spinning of a dimer structure formed by two fluorescent polystyrene particles (1 μm in diameter).

Video S7: Out-of-plane spinning of an inhomogeneously labeled spherical fluorescent microsphere (10 μm in diameter).

Video S8: "Stop-and-go" out-of-plane rotational control of a single fluorescent polystyrene particle (1 μm in diameter).

Video S9: Out-of-plane rotation of three representative assembled microstructures composed of individual particles with a diameter of 4 μm.

Video S10: Peeling a single micro-tile from a two-photon-polymerized micro-tile array.

Video S11: Demonstration of multimodal out-of-plane rotational control of a single micro-tile.

Video S12: Out-of-plane rotation of a budding yeast cell.

Video S13: Out-of-plane rotational and translational motion of fluorescent particles induced by helical TVFs that propagate along circular trajectories.

Supplementary Figures

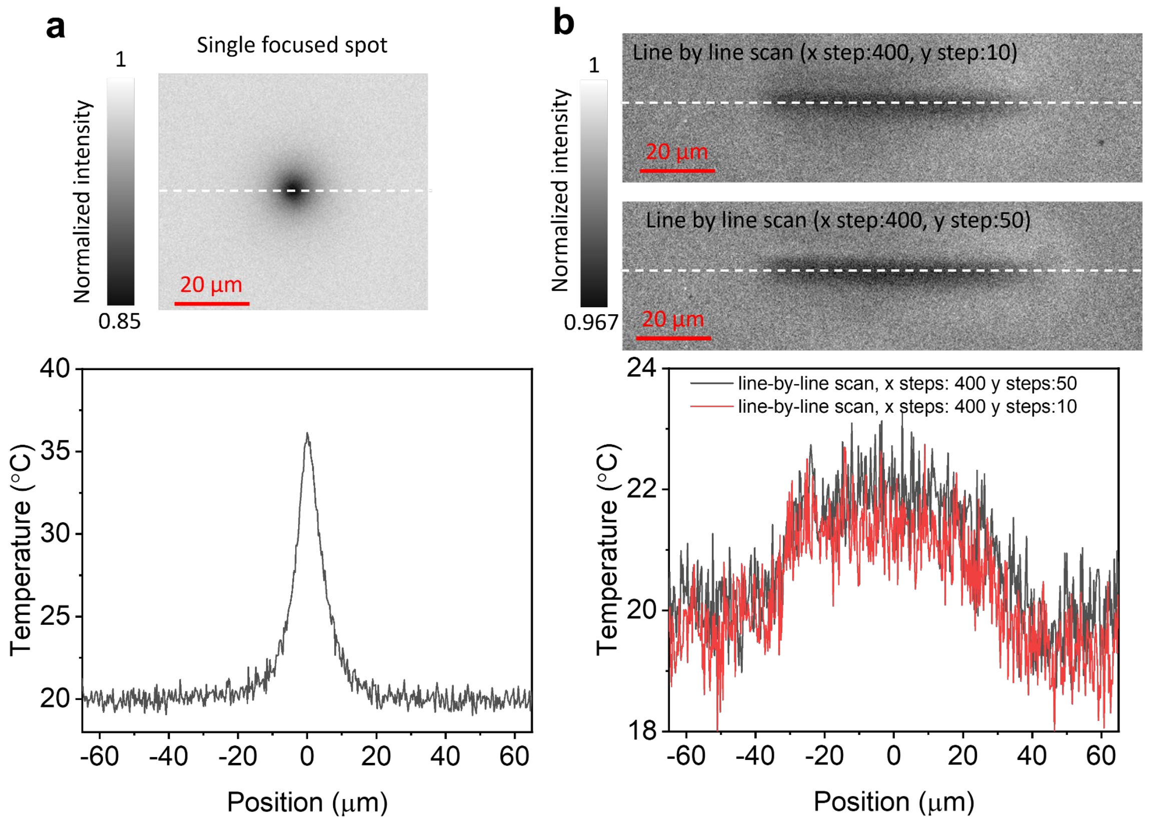

Figure S1. Measured temperature map. (a) Temperature profile of a single focused laser spot. (b) Temperature profile under conditions of line-by-line laser scanning (The scan area spans approximately 68 µm in the *x*-direction and 7.6 µm in the *y*-direction). We use a fixed number of 400 scan steps in the *x*-direction, while the number of scan steps in the *y* direction is changed from 10 to 50. The laser power is fixed at 25 mW. Temperature measurements were performed using fluorescence thermometry based on the fluorescence intensity of Rhodamine B[1]. Specifically, 0.8 mL of the high-viscosity medium was mixed with 15 µL of a Rhodamine B solution (0.2% in isopropanol, 02558-100 ML, Sigma-Aldrich). The fluorescence emission of the medium (mixed with Rhodamine B) was first measured at different controlled temperatures to obtain a calibration curve. The relative emission intensity of the Rhodamine B dye was found to vary linearly with temperature, with a temperature coefficient of -0.0115 K^{-1} for the specific excitation and detection optics.

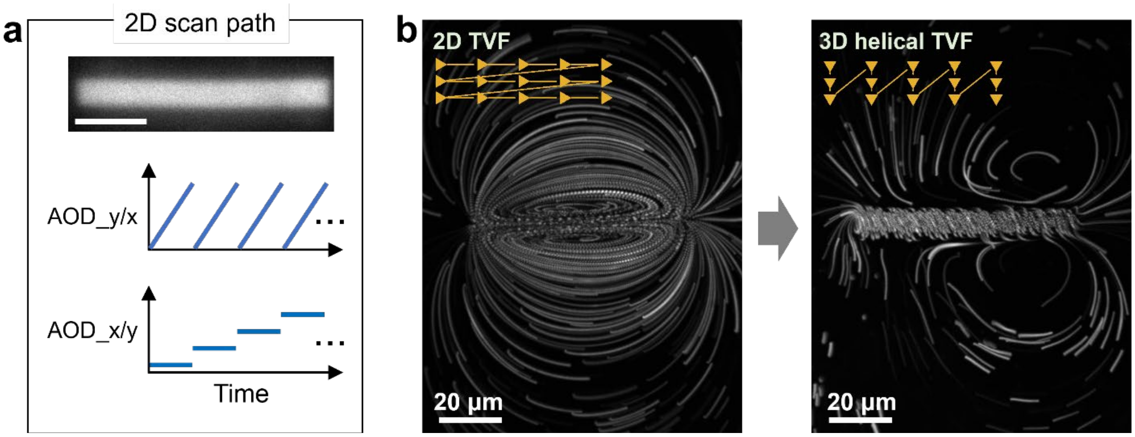

Figure S2. Transformation to 3D helical thermoviscous flows through the use of engineered 2D laser scanning trajectories. (a) Schematic shows the design of two distinct laser scanning sequences, both with the same overall repetition rate and scan range. The scale bar is 20 μm. (b) While both sequences induce flows in the *x* and *y* directions, the flow strengths differ, leading to distinct interplays between orthogonal components. A scanning sequence where the *x*-direction is scanned first, followed by the *y*-direction (i.e., $x \rightarrow y \rightarrow x \rightarrow y$), results in a flow pattern that is fundamentally different from one that begins with *y*-direction scanning (i.e., $y \rightarrow x \rightarrow y \rightarrow x$). Initiating the scanning sequence along the *y*-direction generates a pronounced 3D flow profile. The flow field is visualized by tracking multiple probe particles (500 nm in diameter) through maximum intensity projections over time.

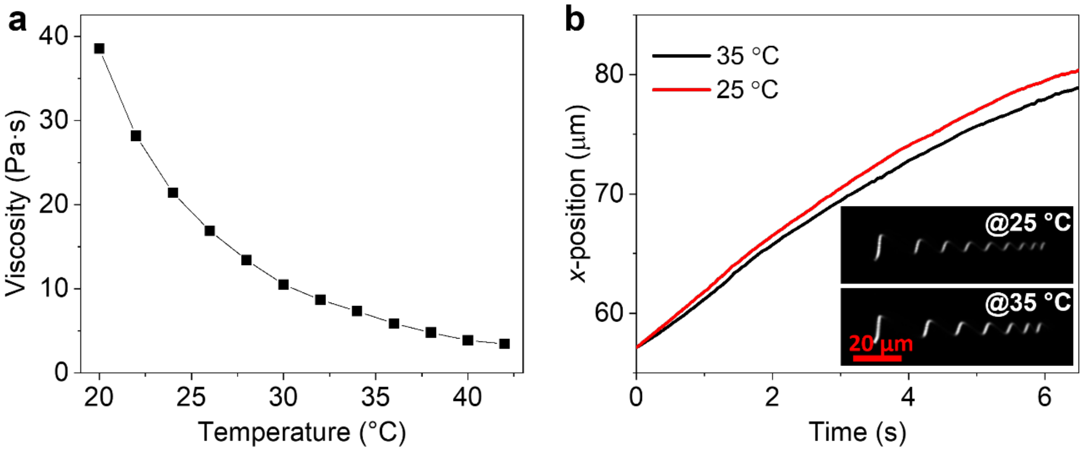

Figure S3. Sensitivity of helical TVFs. (a) Measured viscosity of the medium as a function of temperature. (b) Measured velocity of a single particle along the *x*-axis at two different ambient temperatures. The inset shows the maximum intensity projections of a single fluorescent particle (dia. 1 μm) driven by the TVFs. The laser power and scanning parameters are identical in both cases. The TVFs strictly require a viscosity (η) change with temperature, and there exists a linear dependence between the velocity of the net flows and $d\eta/dT$. For the sugar solutions used, the viscosity-temperature relationship is nonlinear rather than following a simple linear dependence. As a result, the effective sensitivity of the helical TVFs is governed by the relative rate of change of viscosity with respect to temperature, leading to slightly but significantly different helical TVFs at different ambient temperatures even under identical laser scanning parameters.

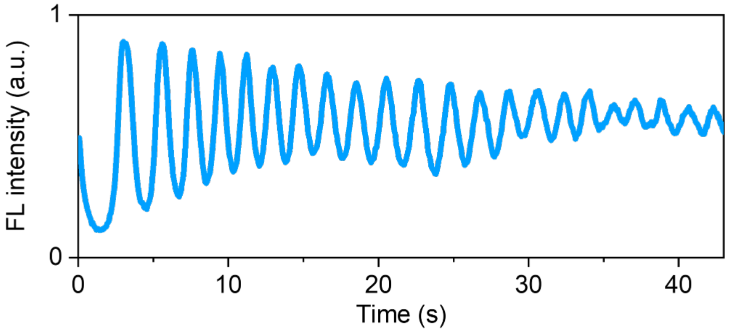

Figure S4. Measured fluorescence intensity of the particle corresponding to Figure 2c (bottom panel).

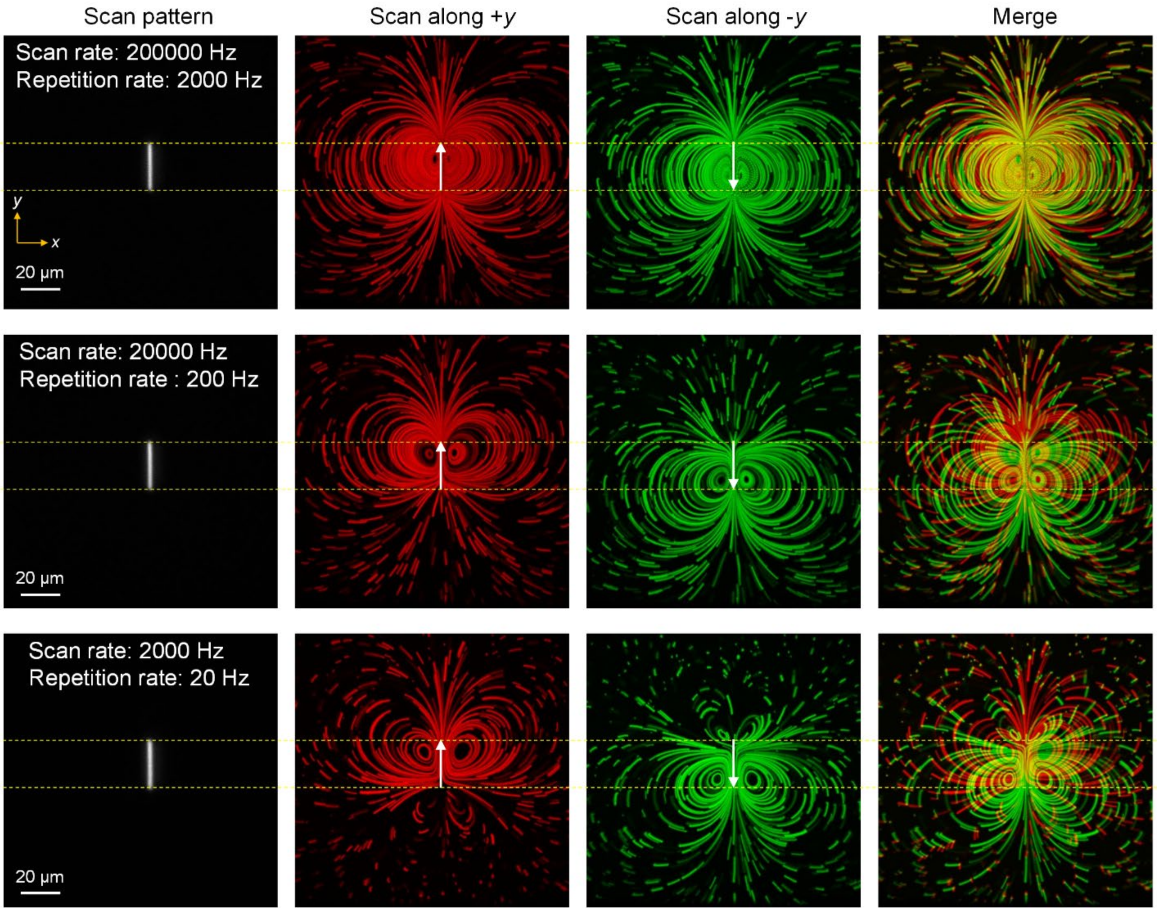

Figure S5. Measured TVFs under different scanning rates. A single repeated line is known to produce an asymmetric flow profile[1] along $\pm y$: scans in the $+y$ direction shifted the flow center toward $+y$, while scans in the $-y$ direction shifted it toward $-y$. These asymmetries are most evident at slower scanning speeds, and less strongly pronounced for higher speeds as used in this work. Likely this shift of the "flow center" depending on scanning direction is related to asymmetry in the temperature distribution induced by the laser heating, in particular, in temperature simulations[2], the temperature field shows asymmetry over the scan period, e.g. its amplitude peaks after the halfway point. From top to bottom, the panels correspond to scanning rates of 200 kHz, 20 kHz, and 2 kHz, respectively.

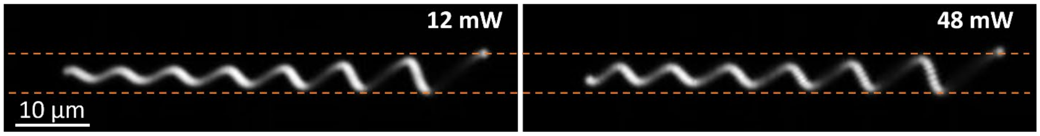

Figure S6. Maximum intensity projections over time of a single fluorescent particle (PS beads, 1 µm diameter) driven by the helical TVFs. These measurements are under the same line-by-line (zigzag) scanning sequence but different laser powers. The left image corresponds to a laser power of 12 mW, and the right image to 48 mW.

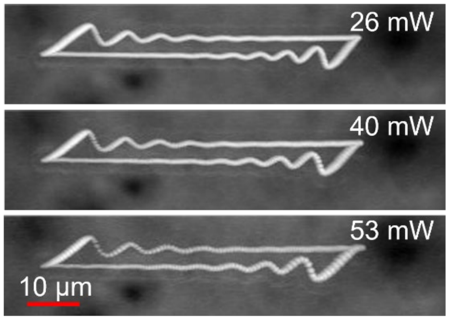

Figure S7. Maximum intensity projections over time of a SiO_2 bead (diameter 1.8 µm) driven by the TVFs. These measurements are under different laser powers with the laser scan sequence the same as that used in Figure 2c (top panel). The top, middle, and bottom images correspond to laser powers of 26 mW, 40 mW, and 53 mW, respectively. Increasing laser power enhances the strength of the TVFs, leading to progressively faster particle motion.

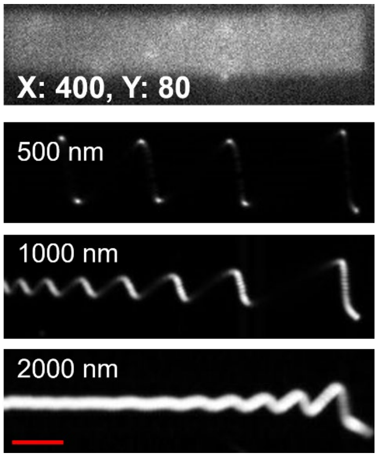

Figure S8. Size-dependent hydrodynamic focusing of fluorescent particles driven by TVFs. The top panel shows the averaged intensity profile of the scanning laser spot, visualized using a layer of upconversion nanoparticles, scanned along a zigzag trajectory directed toward the -*y* and +*x* directions. From the second to the fourth panels, maximum intensity projections over time of fluorescent particles with different diameters reveal a pronounced size dependence in the focusing behavior: smaller particles follow broader trajectories, whereas larger particles are confined more tightly along the central flow axis. The scale bar is 10 µm.

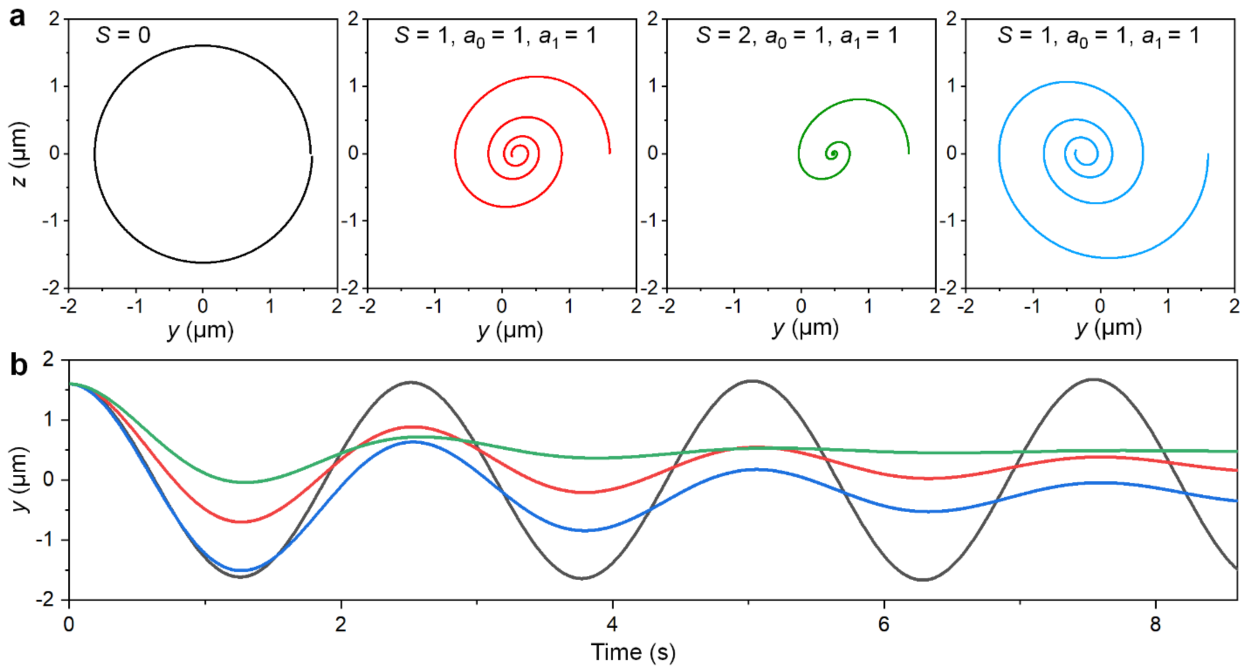

Figure S9. Phenomenological model for hydrodynamic focusing of particle. (a) Calculated particle trajectories in the *y*-*z* plane for different values of the force parameters (S, a_0, a_1), indicated by different colors. The black curve shows a streamline of the background rotational flow in the absence of an external force, for which the particle follows a closed circular orbit. When a bias force along the *z*-direction is introduced, the closed orbit is broken and the particle exhibits spiral trajectories that converge toward a stable focusing position. Increasing the parameter S enhances the magnitude of the external force acting on the particle, mimicking the effect of increasing particle size. The blue trajectory corresponds to a reversed angular velocity, representing a reversal of the laser scanning direction; in this case, the focusing center is shifted toward negative *y*. (b) Time evolution of the particle *y*-coordinate corresponding to the trajectories shown in (a), illustrating the convergence toward a steady-state lateral position. For numerical integration of the overdamped equations of motion, we choose ω_0 = 2.5 rad/s, a fixed time step of Δt = 2 ms, and a particle mobility of μ = 0.6 μm/(pN·s). The particle is initially placed at position (y, z) = (1.6 μm, 0).

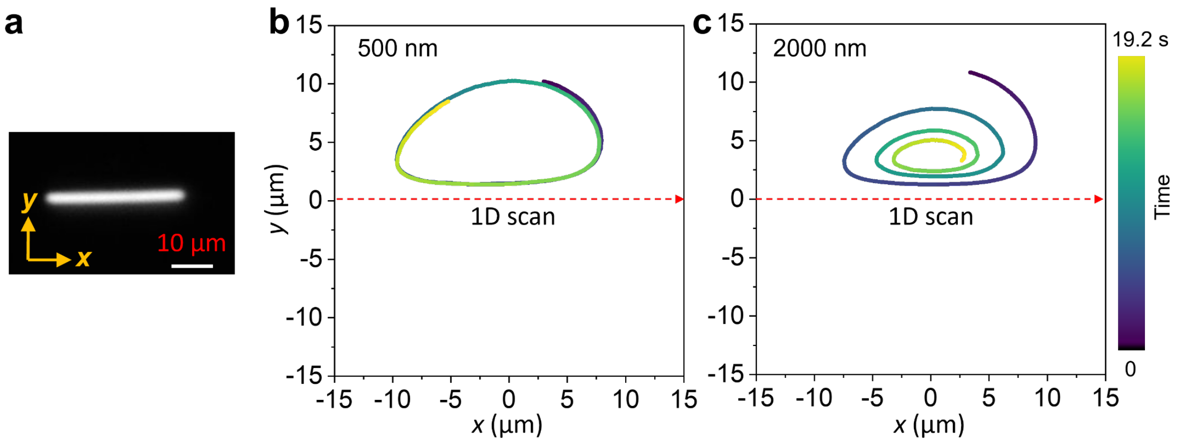

Figure S10. Size-dependent hydrodynamic focusing of fluorescent particles driven by TVFs generated by one-dimensional (1D) line scanning. (a) Averaged intensity profile of the fast-scanning laser spot forming a line shape, visualized using a layer of upconversion nanoparticles. The laser spot is scanned along $+x$ direction. (b) Measured trajectory of a single particle with a diameter of 500 nm, showing motion along a nearly fixed orbit close to the laser scan path (indicated by the dashed red line). (c) Measured trajectory of a single particle with a diameter of 2000 nm, which no longer follows a fixed orbit near the scan path but instead exhibits damped, spiral-like motion similar to that observed in Figure 2.

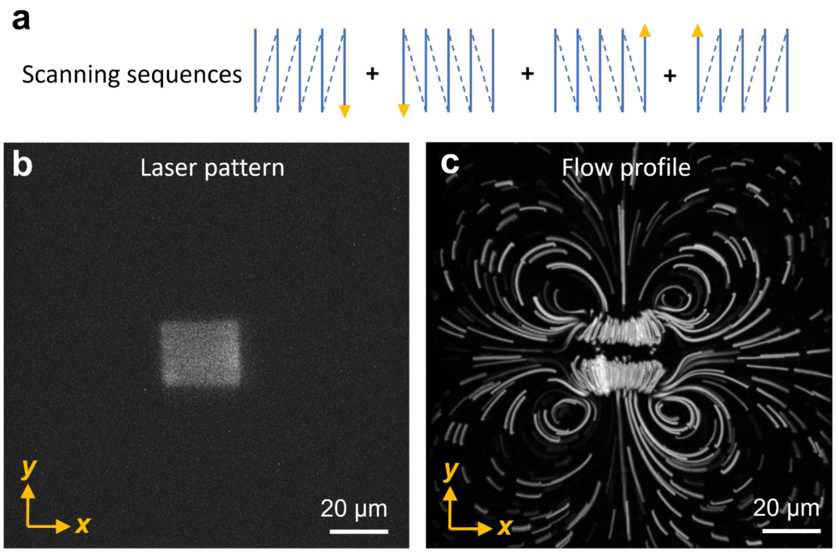

Figure S11. Simultaneous out-of-plane rotations with opposite directions induced by TVFs. (a) Schematic illustrating the laser scanning sequence. (b) Averaged intensity profile of the fast-scanning laser spot forming a two-dimensional rectangular pattern, visualized using a layer of upconversion nanoparticles. (c) Maximum intensity projections over time of fluorescent particles reveal strong out-of-plane rotational TVFs, confined near the upper and lower optical boundaries.

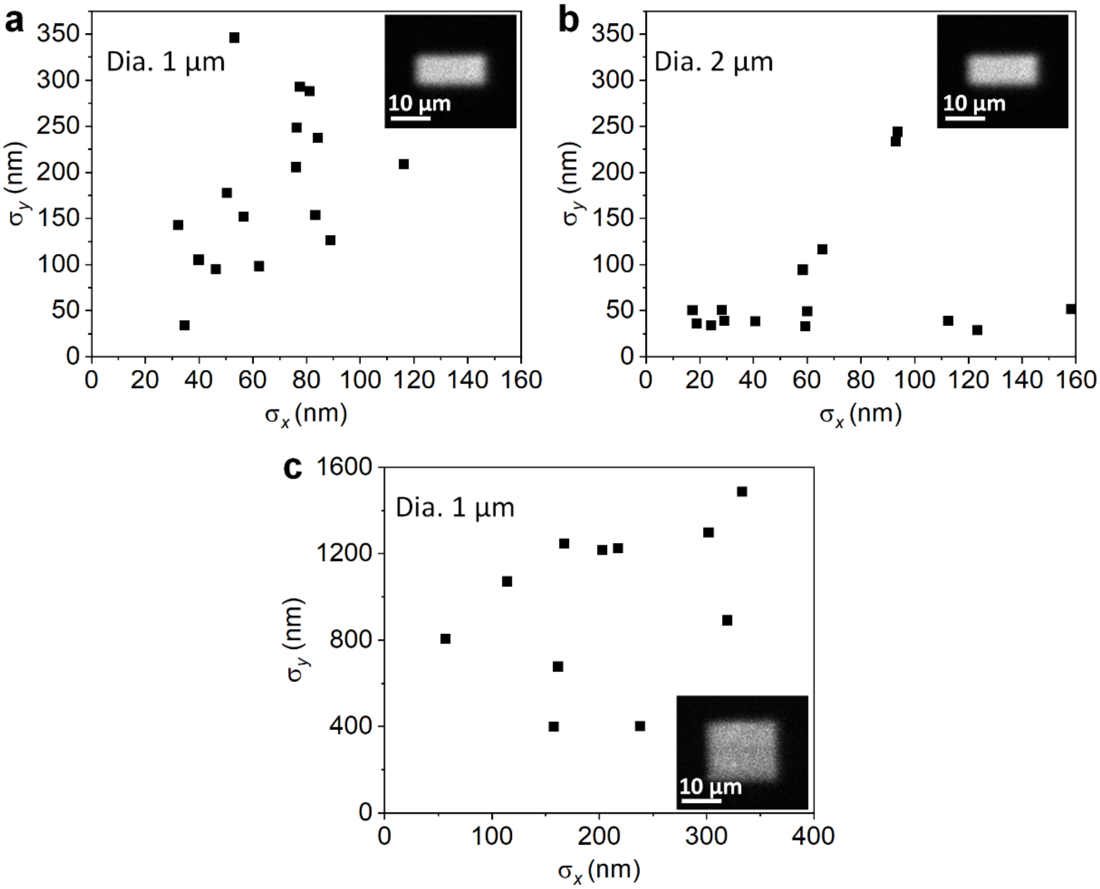

Figure S12. Positional stability of out-of-plane rotated microparticles. Positional fluctuations measured for multiple polystyrene spheres with diameters of (a) 1 µm and (b) 2 µm. Each data point represents an individual particle. Particle trajectories were recorded over time, from which the positional fluctuations along the *x*- and *y*-directions were extracted. The beam scanning sequence is identical to that shown in Fig. 3a, enabling simultaneous stabilization of both *x* and *y* positions. The scan parameters were fixed at 100 steps along the *x*-direction and 50 steps along the *y* direction. The laser power was maintained at 25 mW, and the scan rate was fixed at 500 kHz. Insets show the corresponding laser scan profiles used in the experiments. (c) Effect of increasing the *y*-direction scanning range while keeping the number of scan steps constant (the diameter of the particle is 1 µm), leading to a degradation of the hydrodynamic confinement, as evidenced by increased positional fluctuations.

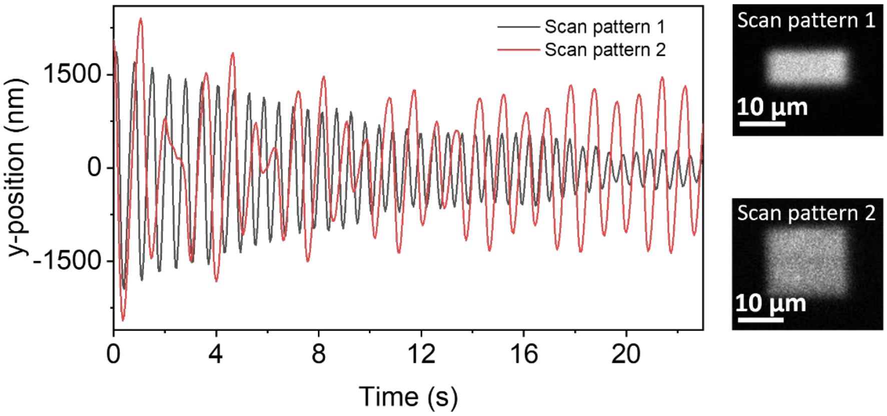

Figure S13. Tunable hydrodynamic focusing via adjustment of the laser scan range. Measured *y*-position of a 1 µm polystyrene sphere under two different laser scan ranges. The scan parameters were fixed at 100 steps along the *x*-direction and 50 steps along the *y*-direction. The laser power was maintained at 25 mW, and the scan rate was fixed at 500 kHz.

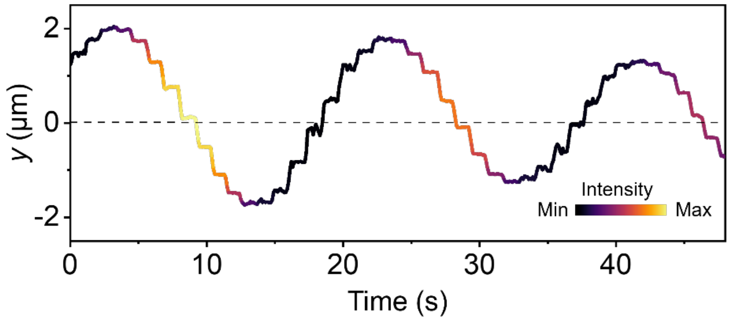

Figure S14. Measured *y*-position of a single PS bead (1 µm in dia.) demonstrating that a temporal delay in the scanning sequence discretizes the rotation, enabling stepwise control analogous to a stepper motor.

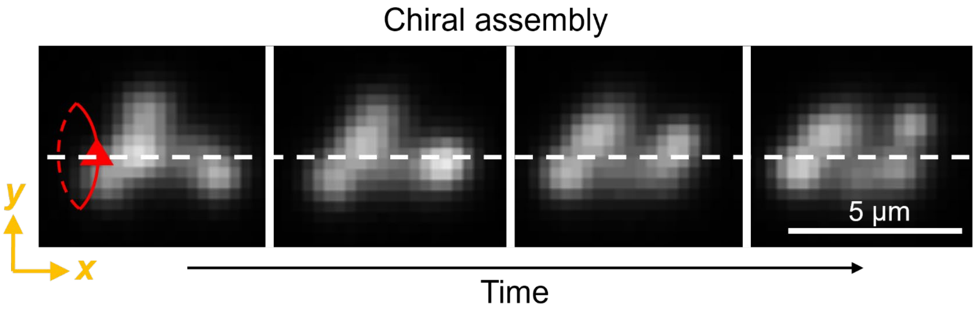

Figure S15. Confocal fluorescence images of assemblies composed of multiple polystyrene beads at various orientations reveal distinct chiral configurations. The structure is rotated around and translated along the *x*-axis, illustrating the three-dimensional arrangement of the assemblies, where the relative positioning of the constituent beads gives rise to handed (chiral) geometries. These results demonstrate the capability of helical TVF-assisted micromanipulation to achieve controlled three-dimensional assembly and orientation of microscale chiral structures.

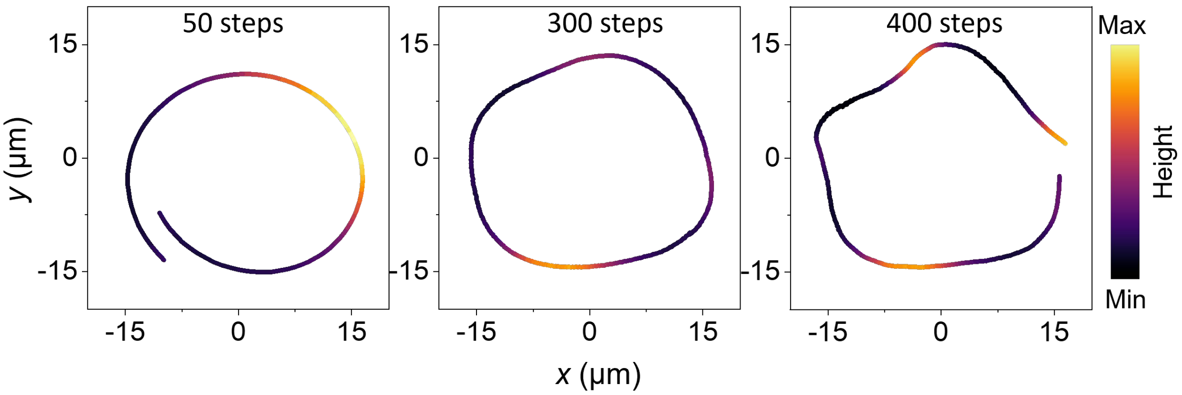

Figure S16. Measured trajectories of a single particle under circular line-by-line laser scanning. The laser beam is scanned azimuthally in 180 steps, forming a circular trajectory. The number of radial scanning steps is 50, 300, and 400 for the left, middle, and right panels, respectively. The laser scanning rate is 500 kHz.

1 Minopoli, A. *et al.* ISO-FLUCS: symmetrization of optofluidic manipulations in quasi-isothermal micro-environments. *eLight* **3**, 16 (2023).

2 Liao, W. & Lauga, E. Axisymmetric thermoviscous and thermal expansion flows for microfluidics. *J. Eng. Math.* **152**, 6 (2025).
